# Supplementary material for: Improvement of the Collection, Maintenance, and Analysis of Neoplastic Cells from Urine Specimens with the Use of CytoMatrix
Source: Methods Protoc. 2021 Sep 10;4(3):65. doi: 10.3390/mps4030065 (PMC8482097; doi:10.3390/mps4030065)
Supplement: Supplementary file 1 [file mps-04-00065-s001.zip › mps-1351010-supplementary.pdf]

methods and protocols

Supplementary Files

	Step	Reagents	Time/Temperature
**1**	Dewax	BOND™ Dewax Solution, 100% Alcohol, BOND™ Wash Solution	Pre-programmed Leica® BOND™
**2**	Antigen Retrieval	Epitope Retrieval Solution 1 (citrate pH 6.0)	20 min., 100˚C
**3**	Peroxide Block	Refine Detection Kit Peroxide Block*	5 min RT
**4**	WASH	BOND™ Wash Solution	3x 2 min RT
**5**	Primary Antibody	Ready-to-Use Primary Antibody for Ki67, CK20 and p53	30 min RT
**6**	WASH	BOND™ Wash Solution	3x 2:00 min.
**7**	Post Primary Mouse Linker	Refine Detection Kit Post Primary*	10 min RT
**8**	Secondary Detection	Refine Detection Kit Polymer*	10 min RT
**9**	WASH	BOND™ Wash Solution/Deionized Water	3 x 2 min RT
**10**	Visualization	Refine Detection Kit Mixed DAB Refine*	10 min RT
**11**	WASH	Deionized Water	3x 2 min RT
**12**	Counterstain	Refine Detection Kit Hematoxylin*	5 min RT
**13**	WASH	Deionized Water	3x 2 min RT
**14**	WASH	BOND™ Wash Solution	3x 2 min RT
**15**	WASH	Deionized Water	3x 2 min RT
**16**	Dehydration (Offline)	Incubate sections in 95% ethanol two times for 10 seconds each. Repeat in 100% ethanol, two times for 10 seconds	
**17**	Clear and Mount co-verslips (Offline)	Incubate sections in xylene two times for 1 minute each. Mount sections with coverslips	

Table S1: Description of the IHC Leica Bond III Protocol, with steps and conditions used for the antibodies.*Reagent included in BOND™ Polymer Refine Detection Kit (Catalog No: DS9800).

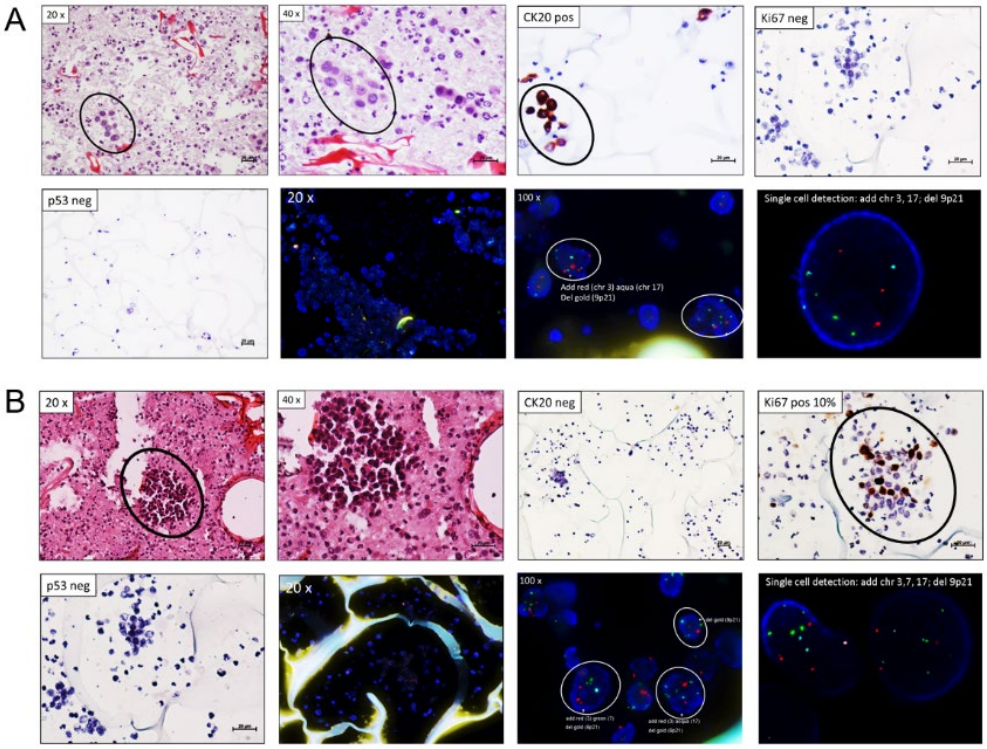

Figure S1: Two representative cases (A and B) diagnosed as HGUC by cytology used for CytoMatrix assay. **(A)** H&E of the urine sample from CytoMatrix-FFPE block N° 1 shows the presence of neoplastic cells (circle in black) with large nucleus, increased nucleus/cytoplasm ratio, nuclear pleomorphism, irregular chromatin and prominent nucleoli (at magnification 20x and 40x). IHC for CK20 shows positive cells with cytoplasmatic/membrane staining, while Ki67 and p53 are negative (at magnification 40x). UroVysion FISH shows the presence of 3 or more signals for chromosome 3 (red) and 17 (aqua) in selected cells (circle in white), with simultaneous homozygous loss of the 9p21 (gold). **(B)** H&E of the urine sample from CytoMatrix-FFPE block N° 4 shows the presence of neoplastic cells (circle in black) with large and hyperchromatic nucleus, increased nucleus/cytoplasm ratio, and nuclear pleomorphism (at magnification 20x and 40x). Neoplastic cells show Ki67 immuno-positivity in >10% (at magnification 40x), while IHC for CK20 and p53 are negative. UroVysion FISH shows the presence of 3 or more signals for chr 3 (red), chr 7 (green) and chr17 (aqua), and homozygous deletion of the 9p21 (gold) in cells (circle in white) of the selected areas.

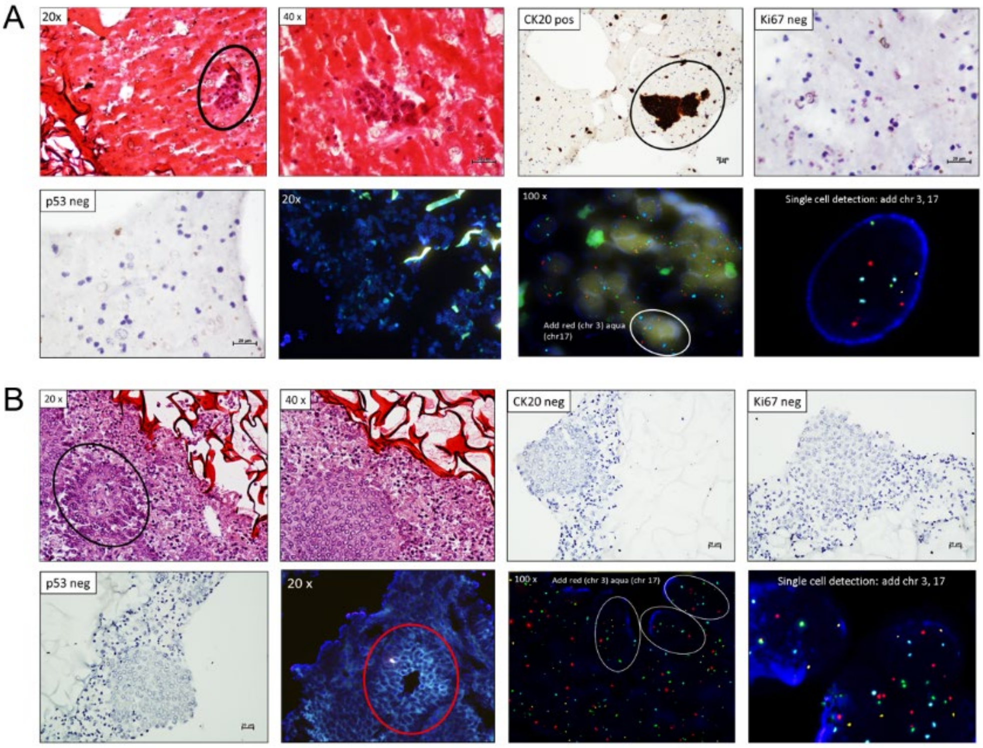

Figure S2: Two representative cases with HGUC (A) and suspected LGUC (B) used for CytoMatrix assay. **(A)** H&E of the urine sample from CytoMatrix-FFPE block N° 3 shows the presence of cells with large and hyperchromatic nucleus, increased nucleus/cytoplasm ratio, and nuclear pleomorphism (circle in black at magnification 20x and 40x). IHC for CK20 shows positive cells (circle in black) with cytoplasmatic/membrane staining; Ki67 and p53 are negative (at magnification 40x). UroVysion FISH shows the presence of nuclei with the presence of 3 or more signals for chr 3 (red) and chr17 (aqua) in around 10% of cells (circle in white). **(B)** H&E of the urine sample from CytoMatrix-FFPE block N° 5 shows the presence of papillary clusters (circle in black), small vessels and a fibrous-connective axis (at magnification 20x and 40x), indicative of LGUC. IHC for CK20, Ki67 and p53 are negative (at magnification 20x). UroVysion FISH shows the presence of neoplastic nuclei counterstained with DAPI in the area of interest (circle in red) at magnification 20x; images at 100x evidence the presence of 3 or more signals for chr 3 (red) and chr17 (aqua) in >10% cells.

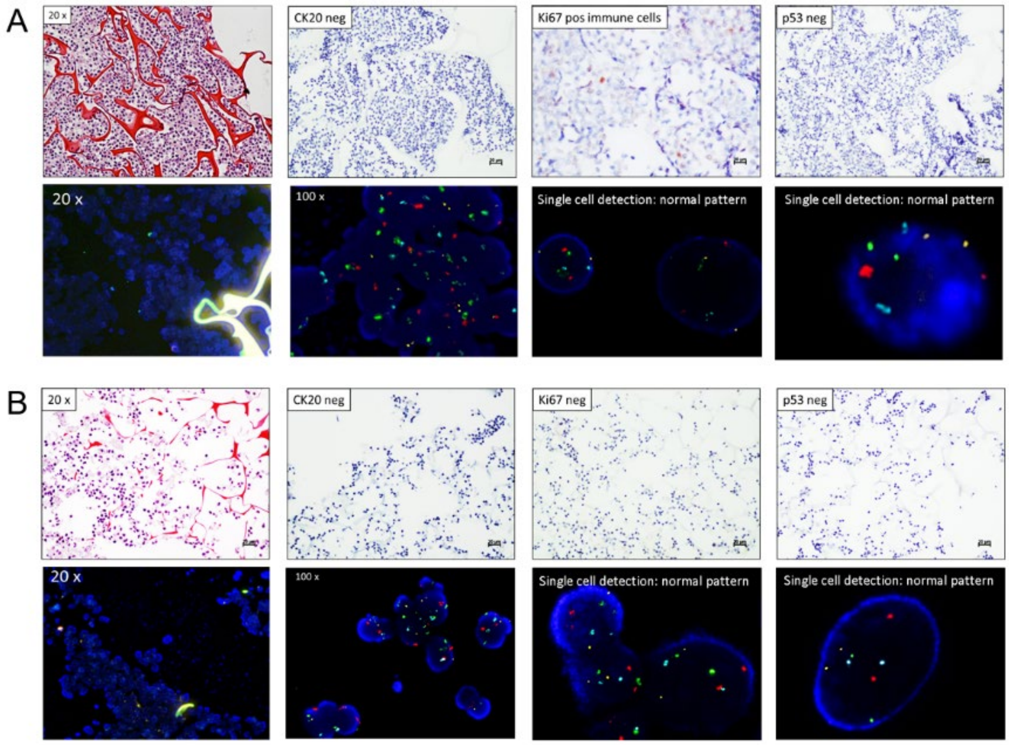

Figure S3: Two representative cases (A and B) negative for urothelial carcinoma by cytology used for CytoMatrix assay. H&E of these urine samples from CytoMatrix-FFPE block show the presence of macrophage and neutrophil granulocyte on both samples N° 10 (A) and N° 12 (B), and the absence of neoplastic urothelial cells (at magnification 20x), confirming cytology results. In sample N° 10, IHC images show negativity for CK20 and p53, while Ki67 evidences <5% of positive granulocytes and immune cells; in sample N° 12, CK20, Ki67 and p53 are all negative (at magnification 20x). In both samples, UroVysion FISH shows the presence of nuclei, counterstained with DAPI, with normal copy number of chromosomes 3, 7, 17 and 9p21 in all the analysable cells, confirming the negativity for urothelial carcinoma.
